# Dietary intakes of expeditioners during prolonged sunlight deprivation in polar enviroments do not support bone health

**DOI:** 10.3402/ijch.v74.27965

**Published:** 2015-08-06

**Authors:** Sandra Iuliano, Jeff Ayton

**Affiliations:** 1 Department of Endocrinology, University of Melbourne/Austin Health, West Heidelberg, Australia; 2 Polar Medicine Unit, Australian Antarctic Division, Kingston, Australia

**Keywords:** Antarctic, bone, calcium, diet, nutrition

## Abstract

**Background:**

Early Antarctic expeditions were plagued by nutrient deficiencies, due to lack of fresh food and reliance on preserved foods. Modern Antarctic expeditioners also require provisions to be shipped in, but improved knowledge and storage options ensure foods are nutritionally sound. Despite this, nutritional imbalances are observed.

**Objectives:**

To determine the adequacy of dietary intake of Antarctic expeditioners, with reference to bone health.

**Design:**

Dietary intake was determined on 225 adults (mean age 42±11 years, 16% female) during 12-month deployments at Australian Antarctic stations from 2004 to 2010, using weighed 3-day food records. Nutrient intake was analysed using FoodWorks. Foods were divided into the 5 food groups according to the Australian Guide to Healthy Eating.

**Results:**

Men consumed below the recommended levels [recommended daily intake (RDI)/adequate intakes (AI)] of calcium (79±42% of RDI, p<0.001), magnesium (83±34% of RDI, p<0.001), potassium (86±29% of AI, p<0.001) and fibre (75±30% of AI, p<0.001), and above the upper limit (UL) for sodium (125±48% of UL p<0.001), whereas women consumed below the recommended levels of calcium (68±21% of RDI, p<0.001) and iron (73±37% of RDI, p<0.001). Vitamin D intake is not substantial (<150 IU/d). Men consumed more alcohol than women (18±24 g/d vs. 10±13 g/d, p<0.05), nearer the guideline of ≤20 g/d. Men and women consumed approximately 1 serving of dairy food per day, and 3 of 5 recommended vegetable servings. Discretionary foods were consumed in excess of recommended.

**Conclusions:**

Improving consumption of calcium-rich (dairy) foods better supports bone health during sunlight deprivation. Increasing vegetable intake to recommended levels will increase fibre, potassium and magnesium intakes. The challenge is the logistics of providing these foods throughout the year.

Lack of nutritional knowledge during early expeditions to polar regions resulted in widespread nutrient deficiencies, starvation and death (1,2). Although starvation is no longer a serious concern for modern expeditioners in Antarctica, the physiological demands of extended periods in polar environments may heighten nutritional requirements. For example, prolonged sunlight deprivation results in bone loss, and affects circadian rhythms and sleep patterns; hence, adequate vitamin D, calcium and magnesium are important (3,4). Immunosuppression is well documented in Antarctic expeditioners, so adequate protein, zinc, selenium, iron, copper, vitamins A, C, E, B6, and folic acid levels are required to support immune function (5,6). Increased metabolic activity because of the extreme conditions may increase requirements of B group vitamins (7).

Overt nutrient deficiencies in Antarctic expeditioners are unlikely, but suboptimal nutrient intakes may affect their work performance and/or health. To help ensure optimal health in expeditioners in this extreme environment, the provision of nutritious foods is required, but within the constraints that most food must be shipped in and stored for long periods of time, and there is limited access to fresh produce.

We aimed to determine whether expeditioners spending 12 months in Antarctica consumed foods from the 5 foods groups in the recommended quantities and if self-selected food intake met nutritional requirements based on published standards, with a particular focus on bone health.

## Materials and methods

The study cohort consisted of 225 healthy adults (86% of all eligible staff), recruited from the 4 Australian Antarctic and Subantarctic stations (Mawson, Davis, Casey and Macquarie Island) between 2004 and 2010. Participants were from a variety of work areas (i.e. manual, scientific and administrative). The majority of expeditioners were employed by Australian government agencies and universities and deployed as part of their work to Antarctica or Macquarie Island for the Austral Winter (June through August). Transfer from mainland Australia to Antarctica occurs between October and March (late spring to early autumn). UV exposure is negligible from March to August and marginal on the shouldering months, corresponding to autumn (March through May), winter (June through August), and the commencement of spring (September through November) in the southern hemisphere (8). All participants underwent a comprehensive medical examination prior to departure and completed a standard medical questionnaire. Of female participants (n=37), 4 were postmenopausal, 5 used the oral contraceptive pill, 1 consumed a calcium supplement and 3 reported consuming multivitamins. No males reported vitamin use.

Participants recorded their food intake over 3 days mid-expedition (i.e. June through July, during the Austral Winter). A standardized food diary was provided to each participant that included clear instructions, and example food entries. Each station was equipped with standard kitchen scales accurate to ±2 g (TANITA TLD-650, Illinois, USA) so the majority of meals consumed at the station foods were weighed. Meals were served à la carte, so expeditioners were able to weigh each item as it was added to the plate. When not accessible (i.e. during field trips), food quantities were estimated in household measures. The nutritional programme used to assess the nutritional content of the food diaries is designed to analyse food by weight (g), volume (ml), standard servings (e.g. slice) or household measures such as a standard cup (250 ml), and teaspoons (5 ml) or tablespoons (20 ml). Diaries were checked by station medical practitioners for completeness and reviewed by a qualified nutritionist prior to analysis. Menu and recipes were obtained from station chefs to input for nutritional analyses. All foods were sourced from the contracted suppliers and exported from Hobart, Tasmania, and delivered by ship on annual Austral Summer resupply voyages. Vitamin D fortification is not mandatory in Australia, except in edible oil spreads so dietary sources of vitamin D are limited. Stations also produced limited quantities of hydroponically grown fruits and vegetables to supplement provisions.

Foods were categorized into the 5 food groups according to the Australian Guide to Healthy Eating (AGHE) and were classified as milk and milk products; meat and meat products; fruit, vegetables, and cereal and grain products (Table I). The “discretionary” food category consists of foods such as cakes, biscuits, confectionery, soft drinks and alcoholic beverages (13). Servings of foods consumed by participants were expressed as a proportion of serving sizes that are consistent with the AGHE. Composite foods were separated into main ingredients and categorized within the relevant food group. Comparison of dietary intake to the national averages for macro- and micro-nutrient intakes was made using data from the Australian National Nutrition Survey (14,15). Individual dietary intakes were analysed for macro- and micro-nutrients using FoodWorks (2009, Professional Edition XYRIS Software, Queensland, Australia). Proportion of recommended daily intake (RDI), adequate intakes (AI) and upper limit (UL) for sodium and guideline for alcohol consumption were based on Australian standards (16,17).

**Table I:** Standard serving for each of the food groups based on the Australian Guide to Healthy Eating.

Food group	Servings
Dairy	1 cup milk; ½ cup evaporated milk; 200 g yoghurt; 1 cup custard; 40 g (2 slices) cheese.
Fruit	1 med piece of fruit (apple, mango, mandarin, banana, pear, peach, etc.); 2 small pieces of fruit (apricot, kiwi, plum, fig); 1 cup diced or canned fruit; ½ cup fruit juice, ¼ med melon (rockmelon, honeydew); 4 pieces dried fruit; 1 ½ tablespoon sultanas; 20 grapes or cherries.
Vegetables	1 med potato or yam; ½ med sweet potato; 1 med parsnip; ½ cup dark green leafy vegetables; 1 cup lettuce or salad; ½ cup beans, peas, lentils, zucchini, mushrooms, tomato, capsicum, cucumber, sweetcorn, turnips, swede, sprouts, celery, eggplant, etc.
Meat and meat products	65–100 g cooked meat or chicken; 80–120 g cooked fish fillet; 2 small eggs.
Grains and grain products	2 sliced bread; 1 cup porridge or breakfast cereal; ½ cup muesli; 1 cup cooked pasta, rice; 1 medium bread roll.
Discretionary	1 med piece of plain cake or bun; 1 tablespoon jam or honey; 1 can soft drink or glasses of cordial; 2 standard glasses of alcohol; 3–4 sweet biscuits; 30 g potato crisps; 2 scoops ice-cream; ½ chocolate bar; slice pizza (2 servings); 1 meat pie or pastie (3 servings); 1 tablespoon butter, margarine or oil.

Fasting blood samples were taken by the station medical practitioners at the time of dietary intake collection for 93 expeditioners during the 2004–2005 and 2005–2006 seasons. These samples were analysed for serum 25(OH)D, parathyroid hormone (PTH), N-mid osteocalcin, total procollagen type 1 amino-terminal propeptide and c-terminal telopeptide, with the procedures and the outcomes reported previously (9).

Data are presented as mean±standard deviations. Comparison of intakes to sex- and age-specific RDI's was made using paired t-tests. The RDI and AI were used to determine the proportion (%) of expeditioners meeting requirements. The UL for sodium was applied to indicate those who exceeded the recommended levels. Sex differences in intakes were determined using unpaired t-tests. Sex differences in proportion meeting recommended intakes were compared using the chi-square test; p<0.05 was considered statistically significant but values of p<0.1 are reported to indicate trends. Data were analysed using SPSS for Windows (Version 19.0, SPSS Australasia Ltd, Melbourne, Australia).

Approval for this study was obtained from the Australian Antarctic Division and the Human Research Ethics Committees of Austin Health.

## Results

Men consumed below the recommended intake of calcium (79±42% of RDI, p<0.001), magnesium (83±34% of RDI, p<0.001), potassium (86±29% of AI, p<0.001) and dietary fibre (75±30% of AI, p<0.001) and above the UL for sodium (125±48% of UL p<0.001), whereas women consumed below the recommended intake of calcium (68±21% of RDI, p<0.001) and iron (73±37% of RDI, p<0.001) (Table II). Men consumed more alcohol than women (18±24 g vs. 10±13 g, p<0.05), with a mean intake close to the Australian guideline of not consuming more than 2 standard drinks per day (i.e. 20 g/d of pure alcohol). Men consumed more energy from protein than women (19.2±4% vs. 17.3±3.5%, p<0.01), tended towards less energy from fats (32.6±6.6 vs. 34.8±6.9, p<0.07), but no sex differences were observed for energy from carbohydrates and alcohol (Table II). Women were younger (35.1±8.1 years vs. 42.8±10.8 years, p<0.0001) and had a lower BMI (25.3±3.8 vs. 27.5±3.6, p<0.01) than men, and had mean intakes of micro- and macro-nutrients more in line with the recommended intake levels (Table II).

**Table II:** Characteristics of Australian Antarctic expeditioners, and nutrient intakes relative to the Australian recommended intake levels

	Males n=188	Female n=37
Demographics		
Age (years)	42.8±10.8	35.1±8.1^a^
Height (cm)	177.9±6.8	166.0±8.1^a^
Weight (kg)	87.3±13.7	69.8±12.1^a^
BMI	27.5±3.6	25.1±3.6^a^
Normal/overweight/obese (%)	26/50/24	61/27/2
Macronutrients		
Energy (kJ/d)	9,552±3,141	8,161±1,884^b^
Protein (g/d)	106±38	82±21^a^
(g/kg body weight)	1.2±0.4	1.2±0.4
% energy	19±4	17±4^b^
Carbohydrates (g/d)	246±93	215±69
% energy	43±8	44±10
Fat (g/d)	85±34	77±25
% energy	33±7	35±7
Alcohol (g/d)	18±24	10±13^c^
% energy	5±6	4±5
Dietary fibre (g/d)	23±10	22±10
AI	30	25
% AI	75±30#	88±38^c^
Proportion below AI (%)	76	68
Micronutrients		
Calcium (mg/d)	788±420	694±207
RDI (mg/d)	1,000	1,000
% of RDI	79±42#	68±21#
Proportion below RDI (%)	79	89
Iron (mg/d)	14.8±6.2	11.7±3.5^b^
RDI (mg/d)	8	18
% of RDI	183±72	73±37^a^,#
Proportion below RDI (%)	1	92
Magnesium (mg/d)	354±147	296±84^c^
RDI (mg/d)	420	320
% of RDI	83±34#	94±26
Proportion below RDI (%)	75	70
Niacin (mg/d)	49.5±19.7	36.0±9.0^a^
RDI (mg/d)	16	14
% RDI	303±109	255±68^c^
Proportion below RDI (%)	0	0
Phosphorus (mg/d)	1,714±640	1,364±287^a^
RDI (mg/d)	1,000	1,000
% of RDI	170±60	136±29^a^
Proportion below RDI (%)	8	5
Potassium (mg/d)	3,271±1166	2,723±726^b^
AI (mg/d)	3,800	2,800
% AI	86±30#	97±26
Proportion below RDI (%)	75	65
Riboflavin (mg/d)	2.4±1.4	1.6±0.5^a^
RDI (mg/d)	1.3	1.1
% of RDI	182±105	143±48^c^
Proportion below RDI (%)	18	11
Sodium (mg/d)	2,887±1160	2,312±645^b^
UL (mg/d)	2,300	2,300
% of UL	125±48#	101±28
Proportion above upper limit (%)	68	22
Thiamin (mg/d)	2.0±1.4	1.4±0.7^b^
RDI (mg/d)	1.2	1.1
% RDI	164±105	125±65^c^
Proportion below RDI (%)	21	38
Vitamin A (µg/d)	856±447	811±266
RDI (µg/d)	900	700
% RDI	94±48	116±38^c^
Proportion below RDI (%)	61	30
Vitamin C (mg/d)	146±174	93±69
RDI (mg/d)	45	45
% RDI	319±374	207±153
Proportion below RDI (%)	21	22
Vitamin D (IU/d)	125±49	106±45
RDI (IU/d)	200	200
Proportion below RDI (%)	89	97
Zinc (mg/d)	13.8±6.2	10.9±3.2^b^
RDI (mg/d)	14	8
% RDI	98±39	136±40^a^
Proportion below RDI (%)	62	16

a p<0.001

b p<0.01

c p<0.05; male vs. female.

# p<0.001, *p<0.01, ~p<0.05; relative to recommended intakes (ref). BMI categories – normal: 18.5–24.9, overweight: 25.0–29.9, obese: ≥30. RDI=recommended daily intake, AI=Adequate intake, UL=Upper limit.

On average, both men and women consumed only 1 serving of dairy food per day compared to the recommended 3 servings/day and required 2 more servings of vegetables to achieve the recommended intake level of 5 servings/day. Servings of grain foods were below the recommended level. Meat and fruit servings were consumed in sufficient amounts, whereas discretionary foods were consumed in excess of the recommended levels (Table III).

**Table III:** Comparison of servings of each of the food groups for Australian Antarctic expeditioners to the Australian Guide to Healthy Eating^a^

	Males n=188	Female n=37
Servings of food groups		
Vegetables	3.0±2.0	3.1±2.0
Recommended	5	5
Proportion below (%)	88	95
Fruit	2.0±2.1	2.3±1.8
Recommended	2	2
Proportion below (%)	58	51
Dairy	1.1±0.9	1.0±0.6
Recommended	3	3
Proportion below (%)	96	100
Meat	2.9±1.7	2.6±1.1
Recommended	1	1
Proportion below (%)	5	6
Grains	3.0±1.8	2.4±1.4
Recommended	6–12	4–9
Proportion below (%)^b^	92	84
Extras	6.2±3.6	5.6±3.0
Recommended	0–3	0–2.5
Proportion above (%)^b^	82	86

a From Ref. (35).

b Based on 3 servings of dairy foods daily for males and 2.5 servings daily for females.

## Discussion

Undernutrition did not affect expeditioners in Antarctica, but intakes below the recommended levels were observed for some bone-related nutrients. Historically observed deficiencies in vitamins C and B were not reported; however, intakes misaligned from recommended were observed for calcium, magnesium, potassium, dietary fibre (all below) and sodium (above) for men, and calcium and iron (both below) for women. The best food sources for most of these nutrients are dairy foods and vegetables, which were consumed below the recommended levels. Amongst other health effects of these dietary deficiencies, the observed patterns of intake may compromise skeletal adaptation to polar environments.

Without adequate sunlight exposure, serum vitamin D [25(OH)D] levels decline, potentially resulting in bone loss (9,18,19). We have previously reported that in 93 expeditioners from this current cohort that were involved in a prospective observational study, serum 25(OH)D was significantly reduced relative to baseline (38.0±16.7 vs. 58.8±20.5 nmol/L, p<0.001), PTH was elevated (4.7±2.2 vs. 3.9±1.8 pmol/L, p<0.01) and bone metabolism was significantly accelerated by 12 months (9). Vitamin D augments active absorption of calcium, the main route when calcium intake is low (20). Calcium intakes in both men and women were below the recommended levels and less than the national averages of 946 and 749 mg/d for Australian men and women, respectively (16). Dairy foods, which are the main source of dietary calcium, were also consumed below the national average of 1.5 and 2 servings daily (21). During prolonged sunlight deprivation, without vitamin D supplementation a higher calcium intake may be necessary to compensate for declining serum 25(OH)D levels. Prior studies indicate that during sunlight deprivation, a mean calcium intake <800 mg/d did not prevent the rise in PTH levels in response to declining 25(OH)D levels (9). Relative to food provision, ensuring at least 3 servings of dairy foods are available on the menu is pertinent. Food fortification (i.e. augmenting calcium intake with milk powder added to foods) is an option to improve calcium intake and is feasible due to the long shelf-life of powdered milk.

Magnesium intake was below the recommended level in males and slightly below the age-matched average consumption of between 383 and 393 mg/d for Australian males (aged 25–65 years) (15). Some, but not all, studies have demonstrated positive relationships between dietary magnesium and bone density in men and women (22,23). In the animal model, magnesium deficiency has been associated with reduced bone formation and increased bone resorption and inflammatory cytokines (24). Rude et al. observed reductions in PTH, 1,25-dihydroxy vitamin D (1,25(OH)2D), lower trabecular bone mineral content (BMC) at the distal femur, greater osteoclast numbers, and increased inflammatory markers in rats when provided magnesium at a level equivalent to 50% of the RDI (25). The authors speculate that the skeletal effects of magnesium deficiency, in part, result from impaired PTH secretion, and direct and indirect effects (via PTH) on 1,25(OH)2D production resulting in impaired osteoblast production and reduced calcium absorption. The increased osteoclast activity is speculated to be in response to the heightened inflammatory markers (24). Most dietary magnesium comes from vegetables, such as dark green, leafy vegetables. Other sources include legumes (peas and beans) and whole grains. The shelf life of leafy vegetables is limited so may be available early after deployment, but a greater reliance on frozen and canned vegetables may alter the intake of magnesium later in the year. Providing whole grain products and canned legumes may maintain magnesium intakes as stores of fresh produce deplete.

Both vegetables and dairy foods are good sources of potassium, and intakes for both food groups were below the recommended levels in men (and women). It has been proposed that potassium from plant sources is consumed in a bicarbonate rich milieu, and so reduces the renal acid load, suggesting a skeletal benefit of fruit and vegetable consumption (26). Epidemiological data involving women indicated that those in the lowest quartile for potassium had lower femoral neck and lumbar spine BMD compared to those in the highest quartile (23). Sufficient vegetables (and fruit) are supplied, but their availability is dependent on the shelf life of produce. Fresh soft produce lasts a few weeks, whereas harder produce may keep for 6 months if stored correctly. The majority of vegetables are provided frozen, with some tinned. Most fruits are provided tinned, some are frozen, and dried fruits are also available. In addition, hydroponics is used to grow tomatoes, cucumbers and herbs. Despite the availability of adequate vegetables, mean intakes were below the recommended levels, and reflect intakes akin to adult Australians (14). Given the greater challenge of maintaining optimal bone health in polar environments, intervention to improve vegetable (and dairy) intakes may be warranted. Dietary fibre intake was below the recommended level for men. This may be reflective of the low intake of vegetables and grain foods, and the high intake of discretionary foods, which contain refined and processed foods. Inclusion of higher fibre foods, for example, whole grain products, and ingredients such as wholemeal flour, will improve fibre intakes.

Sodium intake was above the recommended level for males but similar to previous estimates of mean sodium intake in Australian males (27). High sodium intakes increase urinary calcium excretion and so may contribute to a negative calcium balance. Teucher et al. observed in postmenopausal women that calcium balance was negative when calcium intake was low (518 mg/d), regardless of salt intake (3.9 or 11.9 g), but when calcium intake was high (1,284 mg/d), a high salt intake increased urinary calcium excretion (28). The greatest sources of sodium are processed meats, breakfast cereals, crackers, cheese, crispbread, bread, sweet biscuits and canned vegetables (29). The majority of these foods would be available during expeditions given their long shelf lives. The overconsumption of high salt discretionary foods would also contribute to sodium intake. Providing low-salt snack options may assist in reducing sodium intake.

Mean iron intake was below the recommended level for females but in line with the national average for women of 11.9 mg/d (21). The association between iron intake and bone density in women is not definitive; however, severe iron deficiency induced in rats results in reduced femoral and trabecular BMC, density and strength (30–32). Iron is required for enzymes associated with collagen maturation and in the conversion of 25(OH)D to 1,25(OH)2D (33). Therefore, it has been suggested that with iron-deficient anaemia, these iron-dependent enzymes may be affected resulting in suboptimal metabolism of collagen and vitamin D (34). Iron deficiency may further exacerbate an imbalance in vitamin D metabolism, and hence skeletal adaptation to polar environments. Relative to dietary iron, women consumed adequate servings of meat, with red meat available up to 4 times per week (data not shown). When available, green leafy vegetables provide non-haem iron. Providing fortified breakfast cereals, frozen spinach, and canned lentils and beans may help ensure availability of iron throughout the year.

Alcohol intake averaged 1 standard drink daily for women and 1.8 for men and was within the guideline of no more than 2 standard drinks daily (17). Alcohol intake was similar for the average intake reported for Australian men (18 g/d), but slightly higher than that for women (7.5 g/d) (15). Excessive alcohol consumption is associated with decreased bone density, but evidence regarding the skeletal effect of light to moderate alcohol consumption is inconclusive, with some studies reporting reduced fracture risk (35). However, if alcohol consumption is displacing intake of foods beneficial to bone, then the effects are indirect. Discretionary food intake (including alcoholic drink) exceeded recommendations. As baseline weight was maintained ±2 kg (data not shown), energy balance was sustained, and so alcohol consumption may have been providing sufficient energy, but by displacing nutritious foods.

Limitations of the study need to be acknowledged. Food intake was recorded over 3 days, mid-expedition (Austral Winter). Although 3 days is sufficient to determine intake of macro- and some micro-nutrients, it is not known if this time point reflects usual intake and food availability at other times. Also, it was not determined whether intake during deployment is reflective of usual intake when not in Antarctica, as food records were not obtained before or after deployment. However, the physiological and psychological demands in Antarctica pose additional nutritional challenges so while intake levels may be adequate in temperate climates it may not be suitable in polar environments. Moreover, serum measures, apart from vitamin D, PTH and bone metabolic markers, were not taken so nutrient deficiencies in other nutrients could not be confirmed.

The opportunity for vitamin D synthesis is limited in polar environments so the provision of foods that contain nutrients to support the vitamin D/endocrine systems are required. If the appropriate foods are provided, but not consumed, then strategies to improve dietary intake to support bone health requires investigation and implementation. Such interventions to realign intake to the recommended levels during a polar deployment may also serve well in the general population where similar inadequacies in dietary intakes are also observed.

## References

[1] Lewis HE (1972). Medical aspects of polar exploration: sixtieth anniversary of Scott's last expedition. State of knowledge about scurvy in 1911. Proc R Soc Med.

[2] Anthony JC (2011). The importance of eating local: slaughter and scurvy in Antarctic cuisine. Endeavour.

[3] Iuliano-Burns S, Wang XF, Evans A, Bonjour JP, Seeman E (2006). Skeletal benefits from calcium supplementation are limited in children with calcium intakes near 800 mg daily. Osteoporos Int.

[4] Abbasi B, Kimiagar M, Sadeghniiat K, Shirazi MM, Hedayati M, Rashidkhani B (2012). The effect of magnesium supplementation on primary insomnia in elderly: a double-blind placebo-controlled clinical trial. J Res Med Sci.

[5] Francis JL, Gleeson M, Lugg DJ, Clancy RL, Ayton JM, Donovan K (2002). Trends in mucosal immunity in Antarctica during six Australian winter expeditions. Immunol Cell Biol.

[6] Chandra RK (1997). Nutrition and the immune system: an introduction. Am J Clin Nutr.

[7] Askew EW (1995). Environmental and physical stress and nutrient requirements. Am J Clin Nutr.

[8] Pitson GA, Lugg DJ, Roy CR (1996). Effect of seasonal ultraviolet radiation fluctuations on vitamin D homeostasis during an Antarctic expedition. Eur J Appl Physiol Occup Physiol.

[9] Iuliano-Burns S, Wang XF, Ayton J, Jones G, Seeman E (2009). Skeletal and hormonal responses to sunlight deprivation in Antarctic expeditioners. Osteoporos Int.

[10] Parfitt AM, Rao DS, Stanciu J, Villanueva AR, Kleerekoper M, Frame B (1985). Irreversible bone loss in osteomalacia. Comparison of radial photon absorptiometry with iliac bone histomorphometry during treatment. J Clin Invest.

[11] Holick MF (1994). McCollum Award Lecture, 1994: vitamin D – new horizons for the 21st century. Am J Clin Nutr.

[12] Seeman E (2003). Reduced bone formation and increased bone resorption: rational targets for the treatment of osteoporosis. Osteoporos Int.

[13] Kellett E, Smith A, Schmerlaib Y (1998). The Australian Guide to Healthy Eating.

[14] Magarey A, McKean S, Daniels L (2006). Evaluation of fruit and vegetable intakes of Australian adults: the National Nutrition Survey 1995. Aust N Z J Public Health.

[15] Australian Bureau of Statistics (2008). 4802.0 – National Nutrition Survey: selected highlights, Australia, 1995. https://www.abs.gov.au/.

[16] (1999). Nutrient Reference Values for Australia and New Zealand. http://www.nrv.gov.au.

[17] National Health & Medical Research Council (2009). Australian guidelines to reduce health risk from drinking alcohol. https://www.nhmrc.gov.au/health-topics/alcohol-guidelines.

[18] Oliveri B, Zeni S, Lorenzetti MP, Aguilar G, Mautalen C (1999). Effect of one year residence in Antarctica on bone mineral metabolism and body composition. Eur J Clin Nutr.

[19] Yonei T, Hagino H, Katagiri H, Kishimoto H (1999). Bone metabolic changes in Antarctic wintering team members. Bone.

[20] Zittermann A, Scheld K, Stehle P (1998). Seasonal variations in vitamin D status and calcium absorption do not influence bone turnover in young women. Eur J Clin Nutr.

[21] Australian Institute of Health and Welfare (2012). Australia's food and nutrition 2012. http://www.aihw.gov.au/food-and-nutrition.

[22] Tucker KL, Hannan MT, Chen H, Cupples LA, Wilson PW, Kiel DP (1999). Potassium, magnesium, and fruit and vegetable intakes are associated with greater bone mineral density in elderly men and women. Am J Clin Nutr.

[23] New SA, Bolton-Smith C, Grubb DA, Reid DM (1997). Nutritional influences on bone mineral density: a cross-sectional study in premenopausal women. Am J Clin Nutr.

[24] Rude RK, Singer FR, Gruber HE (2009). Skeletal and hormonal effects of magnesium deficiency. J Am Coll Nutr.

[25] Rude RK, Gruber HE, Norton HJ, Wei LY, Frausto A, Kilburn J (2006). Reduction of dietary magnesium by only 50% in the rat disrupts bone and mineral metabolism. Osteoporos Int.

[26] Lanham-New SA (2008). The balance of bone health: tipping the scales in favor of potassium-rich, bicarbonate-rich foods. J Nutr.

[27] Beard TC, Woodward DR, Ball PJ, Hornsby H, von Witt RJ, Dwyer T (1997). The Hobart Salt Study 1995: few meet national sodium intake target. Med J Aust.

[28] Teucher B, Dainty JR, Spinks CA, Majsak-Newman G, Berry DJ, Hoogewerff JA (2008). Sodium and bone health: impact of moderately high and low salt intakes on calcium metabolism in postmenopausal women. J Bone Miner Res.

[29] Maples J, Wills RB, Greenfield H (1982). Sodium and potassium levels in Australian processed foods. Med J Aust.

[30] Harris MM, Houtkooper LB, Stanford VA, Parkhill C, Weber JL, Flint-Wagner H (2003). Dietary iron is associated with bone mineral density in healthy postmenopausal women. J Nutr.

[31] Lee KS, Jang JS, Lee DR, Kim YH, Nam GE, Han BD (2014). Serum ferritin levels are positively associated with bone mineral density in elderly Korean men: the 2008–2010 Korea National Health and Nutrition Examination Surveys. J Bone Miner Metab.

[32] Medeiros DM, Stoecker B, Plattner A, Jennings D, Haub M (2004). Iron deficiency negatively affects vertebrae and femurs of rats independently of energy intake and body weight. J Nutr.

[33] DeLuca HF (1976). Metabolism of vitamin D: current status. Am J Clin Nutr.

[34] Tuderman L, Myllyla R, Kivirikko KI (1977). Mechanism of the prolyl hydroxylase reaction. 1. Role of co-substrates. Eur J Biochem.

[35] Maurel DB, Boisseau N, Benhamou CL, Jaffre C (2012). Alcohol and bone: review of dose effects and mechanisms. Osteoporos Int.

